# Backbone assignment of *E. coli* NfsB and the effects of addition of the cofactor analogue nicotinic acid

**DOI:** 10.1007/s12104-020-09997-w

**Published:** 2021-01-09

**Authors:** Eva I. Hyde, Alex Ka-Wing Chau, Lorna J. Smith

**Affiliations:** 1 School of Biosciences, University of Birmingham, Edgbaston, Birmingham, B15 2TT UK; 2 Department of Chemistry, University of Oxford, Oxford, OX1 3QR UK; 3 Present Address: Legislative Council Complex, Central, Hong Kong

**Keywords:** Nitroreductase, Flavoprotein, Nicotinic acid, Titration

## Abstract

*E. coli* nitroreductase NfsB (also called NfnB) has been studied extensively, largely due to its potential for cancer gene therapy. A homodimeric flavoprotein of 216 residues, it catalyses the reduction of nitroaromatics to cytotoxic hydroxylamines by NADH and NADPH and also the reduction of quinones to hydroxyquinones. Its role in vivo is not known but it is postulated to be involved in reducing oxidative stress. The crystal structures of the wild type protein and several homologues have been determined in the absence and presence of ligands, including nicotinate as a mimic of the headpiece of the nicotinamide cofactors. There is little effect on the overall structure of the protein on binding ligands, but, from the B factors, there appears to be a decrease in mobility of 2 helices near the active site. As a first step towards examining the dynamics of the protein in solution with and without ligand, we have assigned the backbone ^13^C, ^15^N, and ^1^H_N_ resonances of NfsB and examined the effect of the binding of nicotinate on the amide ^15^N, and ^1^H_N_ shifts.

## Biological context

*E. coli* nitroreductase NfsB is a member of a large superfamily of nitroreductases of over 24,000 sequences, with diverse enzymatic activities, that are being studied for rational enzyme design (Akiva et al. 2017). The *E. coli* enzyme (also called NfnB) was initially discovered as it causes bacteria to be sensitive to nitrofuran antibiotics, such as nitrofurantoin and nitrofurazone (McCalla et al. 1978). Nitrofurantoin is still recommended for use against urinary infections, while nitrofurazone was used topically in skin wounds. Little resistance to these antibiotics has developed, despite several decades of use. The sensitivity of bacteria to these nitroaromatics is because nitroreductases catalyse their reduction to highly cytotoxic hydroxylamines, by NADH and NADPH. This reaction is the basis for the potential use of NfsB in cancer gene therapy. Introduction of the *nfsB* gene into cancer cells, for instance by use of a viral vector, followed by treatment with prodrugs such as CB1954 (5-aziridin-1-yl, 2, 4, dinitrobenzamide) has been shown to kill the cells and neighbouring cells (Searle et al. 2004). Similarly, delivery of *nfsB* followed by treatment with metronidazole has been used for selective cell ablation in the study of animal development (Curado et al. 2007). The NfsB homologue from *Enterobacter cloacae*, in turn, has been studied for use in bioremediation of TNT and in chemical transformations (Miller et al. 2018). In addition to reduction of nitroaromatics, NfsB reduces quinones to quinols, in a 2-electron step, without producing radicals. It has been postulated therefore to be involved in reducing oxidative stress. In support of a role in this, its expression is upregulated by the transcription activator MarA (Barbosa and Levy 2002).

**Fig. 1:**
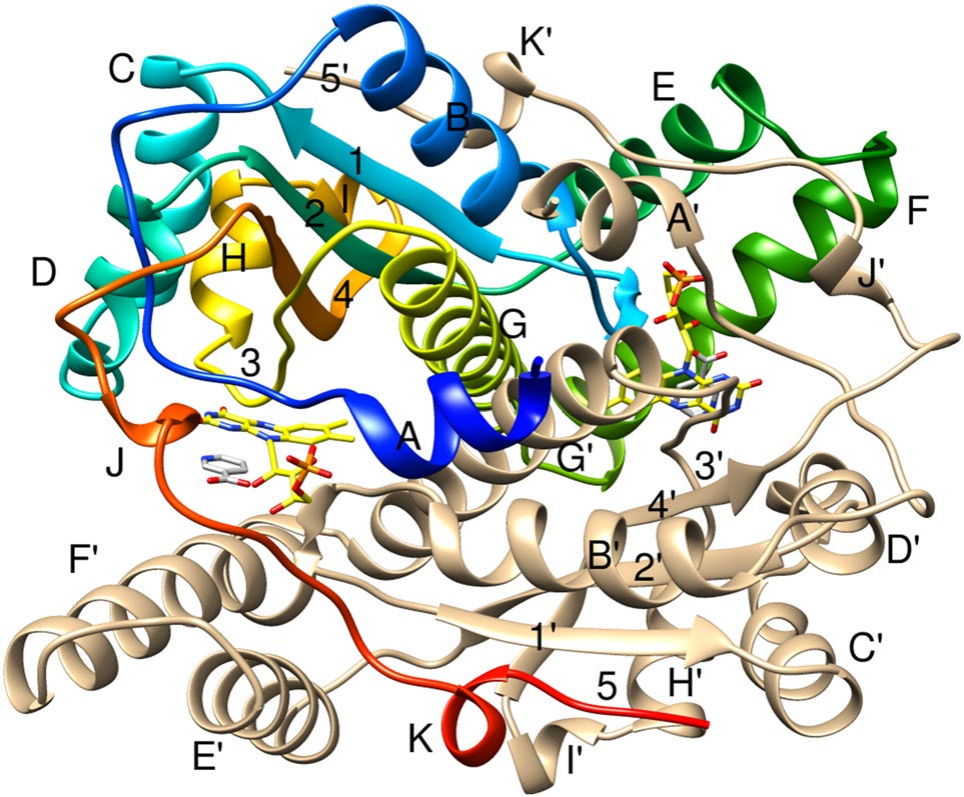
Crystal structure of *E. coli* NfsB bound to nicotinate. Ribbon diagram of the 3D structure of *E. coli* NfsB bound to nicotinate (from 1ICR (Lovering et al. 2001)). One subunit is coloured in rainbow colours, blue to red, from the N-terminus to the C-terminus, with the helices labelled A-K and the strands numbered 1–5. The other subunit is in beige and labelled A′-K′ and 1′-5′. The FMN cofactor and nicotinate are shown as sticks, with FMN carbon backbone in yellow, the nicotinate in grey and other atoms in CPK colours

The *nfsB* gene encodes 217 amino acids, but, like many *E. coli* proteins, the first methionine, residue is cleaved in vivo so the protein contains 216 amino acids per subunit. It is a homodimer with a tightly bound FMN cofactor in each subunit. The crystal structures of the wild type NfsB protein (Lovering et al. 2001; Parkinson et al. 2000), mutants, and several homologues have been determined (Fig. 1). Each subunit contains 11 helices and 5 highly twisted β-strands with an extensive subunit interface. The FMN cofactors lie on opposite sides of the long, G helices, that cross each other, and each cofactor contacts both subunits. There is little effect on the overall structure of the protein on binding ligands, but, from the B factors, there seems to be a decrease in mobility at the end of helix E and throughout helix F, residues 105–132 (Lovering et al. 2001; Parkinson et al. 2000). These two helices protrude from the core of the protein but are near the active site. Protein flexibility has been postulated to be important for the catalytic activity of many enzymes, including the homologous NADH oxidase (NOX) from *Thermus thermophilus*. The NMR relaxation properties of NOX, have been determined (Miletti et al. 2011) and molecular dynamics simulations have compared the dynamics of NOX, a thermophilic enzyme, to those of the mesophilic NfsB as a function of temperature, suggesting again that helices E and F may influence stability and activity (Merkley et al. 2010). There have been other molecular dynamics calculations of *E. coli* NfsB (Christofferson et al. 2012) and the homologous *Enterobacter cloacae* enzyme (Christofferson 2020), to try to resolve the molecular mechanism of nitroaromatic reduction. As a first step towards examining the dynamics of the protein in solution with and without ligand, we have assigned the backbone ^13^C, ^15^N, and ^1^H_N_ resonances of NfsB and examined the effect of the binding of sodium nicotinate, as a mimic of the NAD(P)H headpiece, on the amide ^15^N and ^1^H_N_ shifts.

## Methods and experiments

### Protein expression and purification

Although NfsB expressed well in minimal M9 medium from the *trp*-promoter in plasmid pPM24 in *E. coli* DH5α (Michael et al. 1994), no expression was found when the strain was grown in D_2_O in this medium. The *nfsB* gene was amplified from *E. coli* DH5α and cloned into pET11c, expressed without tags from *E. coli* BL21 in M9 medium, and purified as described previously (Lovering et al. 2001). Prior to the NMR experiments it was dialysed into 20 mM sodium phosphate buffer, pH 7.0, 0.05 mM EDTA. For assignment, the sample was ^13^C/^15^N/^2^D labelled by growth of the *E. coli* in M9 medium containing 1 g/l ^15^N-NH_4_Cl, and 2 g/l ^13^C_6_-glucose as the only nitrogen and carbon sources and 80% D_2_O. For the titration experiments with nicotinic acid, the protein was labelled with ^15^N only. The expressed protein contained FMN but, to maintain full saturation with the cofactor, 20 µM FMN was added to all buffers throughout protein purification, apart from the final dialysis. That the protein was fully bound with FMN was verified by measuring the ratio of absorbance of the protein preparation at 373 nm and 454 nm (where only the FMN absorbs) and at 280 nm (where both FMN and protein absorb).

### NMR spectroscopy

For assignment of the spectrum of the protein in the absence of ligand, HNCOCACB and HNCACB spectra were recorded on a Varian 800 MHz spectrometer at 37 °C. HNCOCA, HNCA, HNCO and HNCACO spectra were taken on a Varian 600 MHz spectrometer at 30 °C or 37 °C, all with the triply labelled protein and deuterium decoupling. Assignments were confirmed where possible, by looking for sequential NH-NH NOEs in a ^15^N-^1^H NOESY-HSQC taken at 600 MHz using a ^15^N-labelled sample.

Spectra were processed with NMRPipe (Delaglio et al. 1995) and analysed with CCPN software (Vranken et al. 2005), or with NMRView5 (Johnson and Blevins 1994) and UCSF SPARKY (Goddard and Kneller 2008).

In the titrations, small aliquots from a stock solution of 89 mM sodium nicotinate in the same buffer as the protein, were added to (0.4 ml) of 0.53 mM protein. 1D ^1^H NMR spectra and 2D ^15^N-^1^H HSQC spectra were taken after each addition, using a Varian 600 MHz spectrometer at 30 °C and, in a separate titration, at 35 °C. The concentration of ligand ranged from 0 to 8.9 mM. ^15^N-^1^H NOESY-HSQC spectra, with a mixing time of 0.1 s, were taken before and after the titration. The spectra were assigned by following the shift changes over the titration at both temperatures and assignments confirmed, where possible, by comparing the ^1^H-^15^N-HSQC NOESY spectra of the protein with and without ligand. The changes in ^15^N shifts between the free protein and protein in the presence of 10 equivalents of ligand were weighted by a factor of 0.15 relative to the ^1^H_N_ shifts as in Mulder et. al (Mulder et al. 1999).

The shifts of each nicotinate proton (y) as a function of ligand concentration (x) were fitted to a simple hyperbola to estimate the bound shift (y0)

1 $$y = y0 + \frac{a*x}{{Kd + x}}$$

where a is the difference between the bound and the free shift and K_d_ is the apparent dissociation constant.

### Extent of assignments and data deposition

#### Free protein

**Fig. 2:**
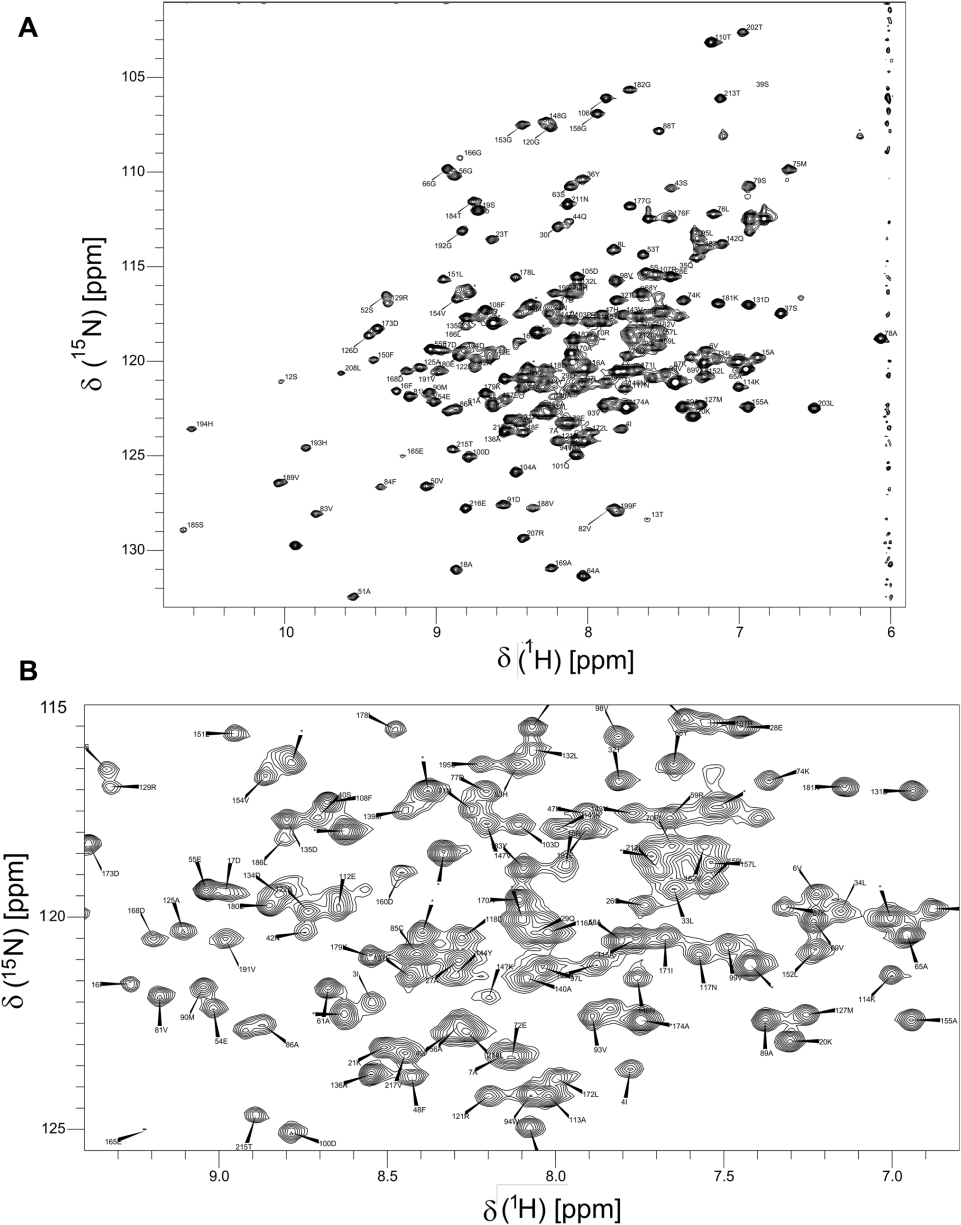
^1^H-^15^N HSQC NMR spectrum of *E. coli* NfsB, taken at 600 MHz and 35 °C, in 20 mM sodium phosphate buffer, pH 7.0, 0.05 mM EDTA. **a**- full spectrum of the amide region, with NH assignments labelled, side chains are not assigned. Asterisks indicate overlapping peaks. **b**- inset of central region of the spectrum, with NH assignments

**Fig. 3:**
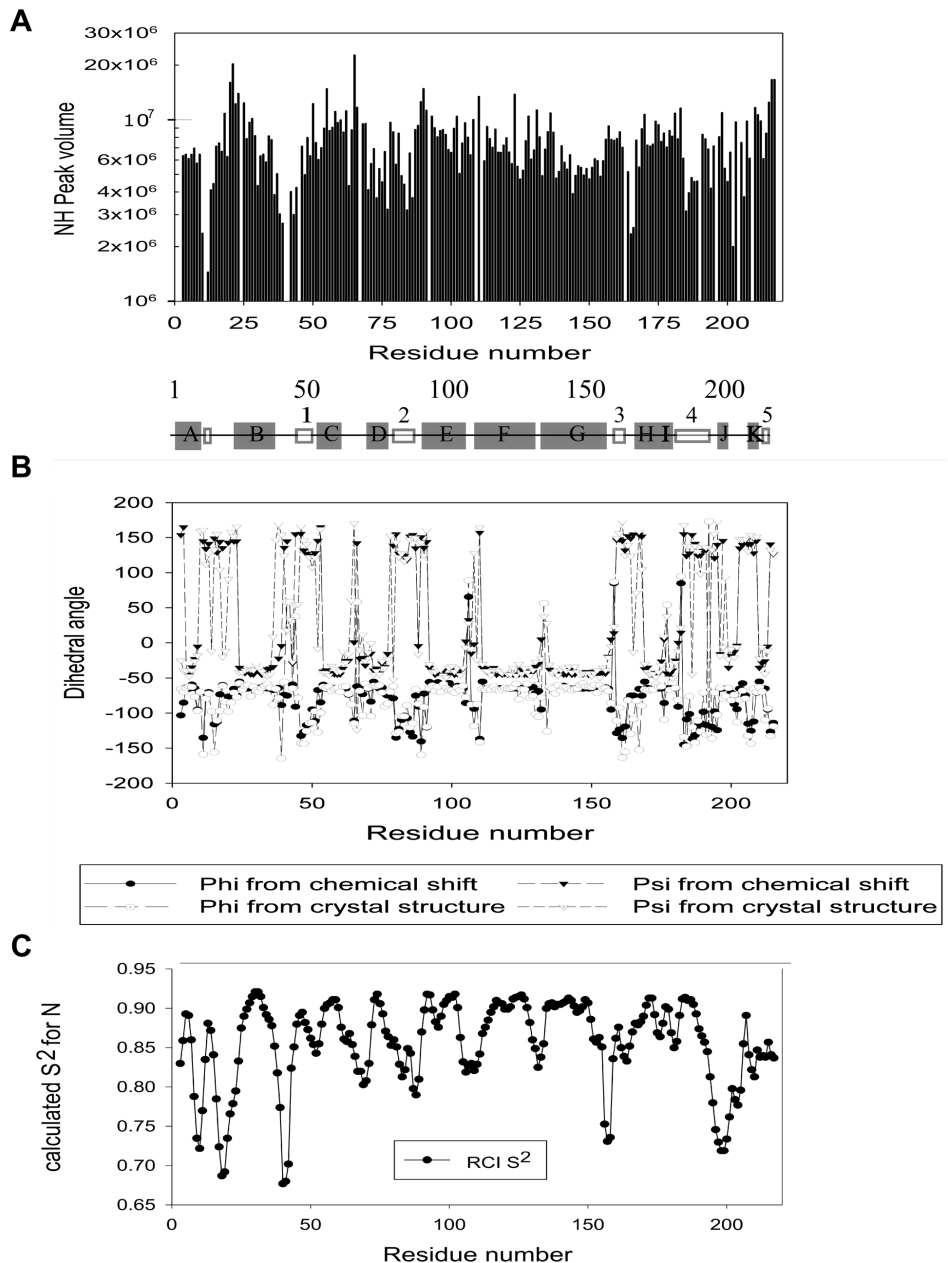
NH Peak volumes, secondary structure and S^2^ values of *E. coli* NfsB, based on the chemical shifts. **a** Relative peak volumes from a 3D HNCO spectrum of *E. coli* NfsB, taken at 600 MHz and 35 °C, in 20 mM sodium phosphate buffer, pH 7.0, 0.05 mM EDTA. Centre- Secondary structure from the X-ray crystal structure of the protein, from 1ICR (Lovering et al. 2001)- helices shown in grey boxes labelled with letters, strands shown in white boxes with numbers. **b** Comparison of secondary structure of the free *E. coli* NfsB determined from the chemical shifts by the program DANGLE (Cheung et al. 2010) and that determined by X-ray crystallography from 1ICR (Lovering et al. 2001). Circles, phi angle, triangles psi angle, grey symbols values from DANGLE, white symbols values from 1ICR. **c** S^2^ values for the NH groups based on the chemical shifts, calculated using the RCI method (Berjanskii and Wishart 2008) in TALOS-N (Shen and Bax 2013)

The expressed protein is a dimer with 216 residues per monomer, of which 9 are proline. The high molecular mass (48 kDa) meant that the protein needed to be deuterated for good signal in triple resonance experiments and the spectra were taken at relatively high temperature to reduce the rotational correlation time. Figure 2 shows the ^1^H-^15^N HSQC spectrum of the protein with the assignments. 200 NH residues were assigned in the triple resonance spectra. NH peaks for the first residue Asp 2 (not expected), and for His 11, Thr 41, Asn 67, Asp 92, Ala 109, and Val 196 were not observed, while some other peaks had very low intensity in the spectra (Fig. 3a). Cα, Cβ and C′ peaks were assigned for all residues. These assignments have been deposited in the BioMagResBank, with ID 50476.

In addition to the backbone resonances, NH peaks were assigned to the indole NH groups of the three tryptophan residues but were not assigned further. These are at 11.80, 135.0; 9.93, 129.8; and 10.96, 130.7 ppm, ^1^H and ^15^N shift, respectively.

His 195 NH, which is hydrogen-bonded to Asp 160, and Ser 185 NH, which is hydrogen-bonded to Asp 163 and close to Phe 167, have ^1^H shifts greater than 10 ppm, and Ser 185 also has a high ^15^N shift. Ala 78 NH is close to Phe 16 which may cause its ^1^H shift close to 6 ppm. Met 75 NH has an unusually low ^15^N shift. It is close to the backbone carbonyl oxygen of both Glu 72 and Arg 73.

The secondary structure of the protein was determined based on the chemical shifts, using DANGLE (Cheung et al. 2010) and agrees well with the crystal structure (1ICR (Lovering et al. 2001)), Fig. 3b. The NH order parameters for the protein were also calculated from the chemical shifts, using the RCI method (Berjanskii and Wishart 2008) in TALOS-N (Shen and Bax 2013), Fig. 3c. Most of the order parameters are above 0.8, apart from a few residues in loops, suggesting that the protein is structurally quite rigid, as found for NOX (Miletti et al. 2011). The calculated order parameters do not suggest greater flexibility of helices E and F as shown by the B factors of the free protein (Parkinson et al. 2000) and by the molecular dynamics simulations (Merkley et al. 2010). Instead the low order parameters, Fig. 3b, seem to correlate with low NH peak intensities, Fig. 3a. This suggests that the weaker intensities for loop NH resonances are due to enhanced fast motion, and thus more NH exchange.

#### Nicotinic acid binding

**Fig. 4:**
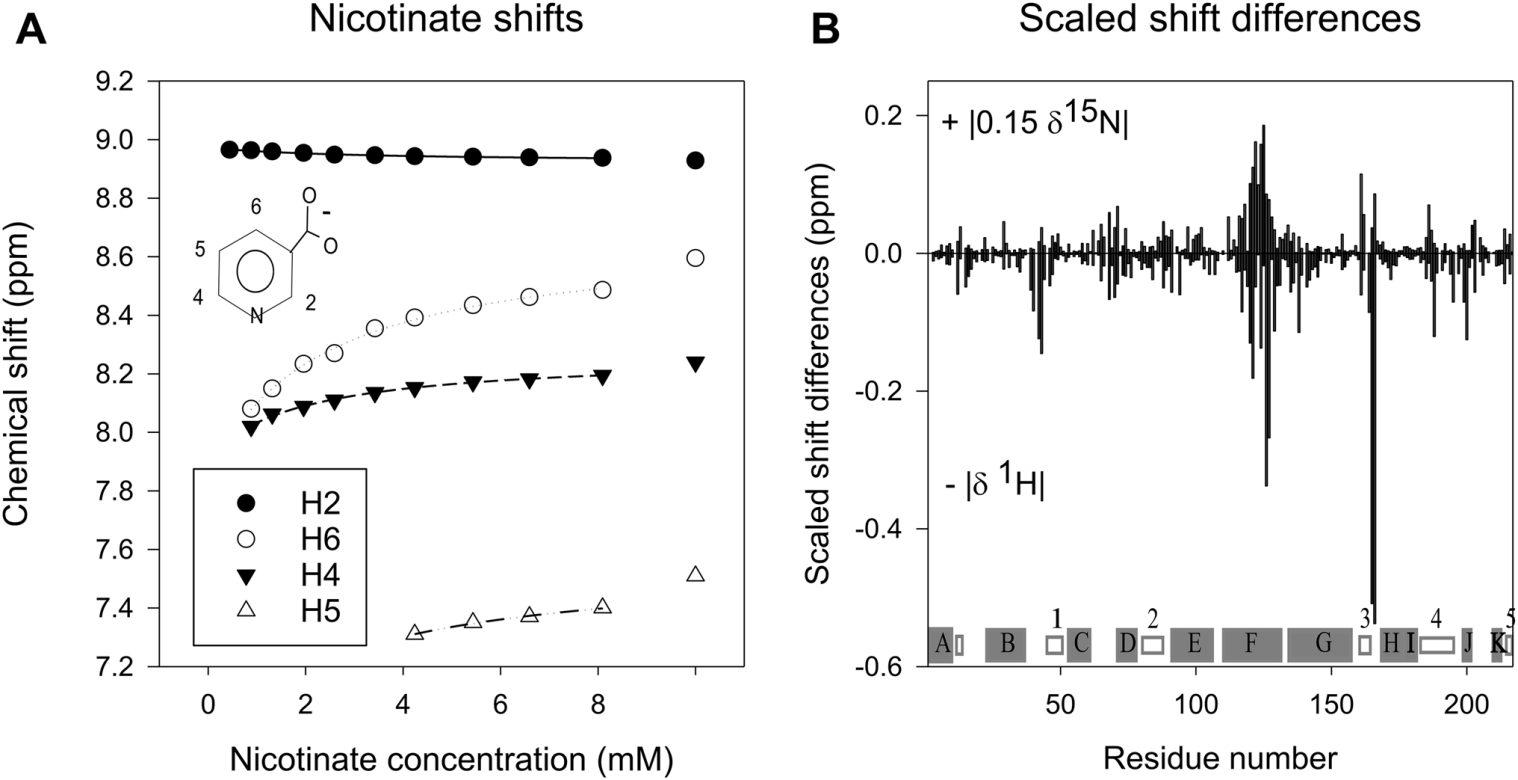
Shifts on binding nicotinate. **a** Chemical shifts of nicotinate on titration into *E. coli* NfsB. The initial protein concentration was 0.53 mM. Aliquots from a stock solution of 89 mM sodium nicotinate in the same buffer as the protein, were added to 0.4 ml protein solution. Shifts were followed by 1D ^1^H NMR spectra after each addition, using a Varian 600 MHz spectrometer at 30 °C. The final symbols are those of the free ligand in the same buffer. Black circles- H2, white circles H6, black inverted triangle H4, white triangle H5. Lines show the fit of the data to hyperbolas (Eq. 1). **b** Changes in NH chemical shifts between free protein and protein containing 10 equivalents of nicotinate. Positive values, absolute value of 0.15 times the difference in ^15^N shift. Negative values: absolute values of changes in the ^1^H shift. Below: secondary structure from 1ICR- helices shown in grey boxes labelled with letter, strands shown in white boxes with numbers

The shifts of the aromatic protons of the ligand were followed in 1D ^1^H NMR spectra taken on titration of nicotinate into ^15^N-labelled NfsB (Fig. 4a, Table 1), while the shifts of the amide groups were followed using ^15^N-^1^H HSQC experiments. (Fig. 4b).

**Table 1:** Estimated bound shifts, and apparent dissociation constants for Nicotinate, using fit of shifts to the equation for a simple hyperbola

Proton	Estimated bound shift	Estimated change in shift on binding	Apparent K_d_
	(ppm)	(ppm)	(mM)
H2	8.87 ± 0.05	0.050 ± 0.003	3.0 ± 0.8
H6	7.84 ± 0.04	0.83 ± 0.02	2.2 ± 0.4
H4	7.92 ± 0.02	0.35 ± 0.01	1.9 ± 0.3
H5	7.2 ± 0.2	0.3 ± 0.1	2.5 ± 4

Three of the proton resonances of the ligand were in fast exchange, while one (H5, a triplet) was severely line-broadened on binding and so only observed at relatively high concentrations of ligand. The nicotinate protons shift to lower frequency on binding, apart from the H2 which shows a slight shift to higher frequency. The nicotinic acid binds parallel to the FMN ring system, and the protons would be expected to be greatly affected by this, as well as by protein contacts.

For the protein, a few NH peaks were not observed and several peaks overlapped either in spectra taken in the absence of ligand, or taken in its presence. Where possible, the assignments of the ligand-bound protein were confirmed by comparing the ^1^H-^15^N-HSQC NOESY spectra of the protein with and without ligand. These assignments have been deposited in the BMRB under ID 50576.

**Fig. 5:**
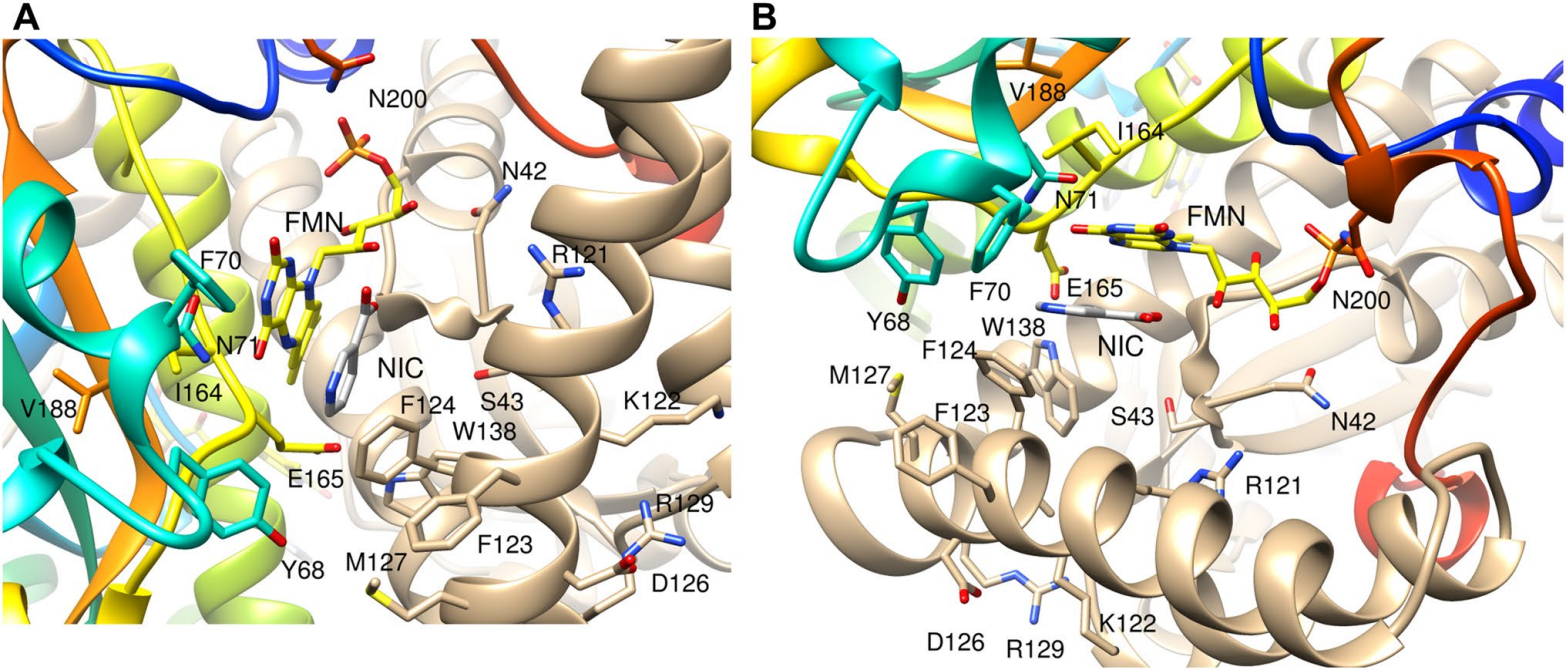
Two views of the structure of *E. coli* NfsB near bound nicotinate, showing the residues with significant changes in chemical shifts on nicotinate binding. Ribbon diagram with the two subunits of the dimer in coloured as in Fig. 1 and the residues affected by nicotinate binding in stick form, labelled by residue. The FMN cofactor is in yellow and CPK colours and labelled, and the nicotinate is in light grey and CPK colours and labelled NIC

Several amide resonances shifted on titration with nicotinate and some changed intensity. Assignments of NH peaks from residues Ser 40, Phe 70, Glu 72, Phe 123, Phe 124, Asp 135 and Val 162 remain tentative, because of peak overlap. The largest overall shift changes were seen at residues 165 and 166, and between residues 120–129. Smaller, but significant, shift changes were observed at Asn 42, Ser 43, Trp 138, Val 188 and Asn 200 (Fig. 4b). The amino acids whose NH shifts were most affected by titration are shown in the crystal structure of the protein complex in Fig. 5. Changes in peak intensity, both increases and decreases, were observed for many resonances across the whole protein, often for amino acids in loops.

Nicotinic acid makes direct contact with Ser 40, Thr 41, Phe 124, Glu165, Gly 166, Leu 203 and the FMN cofactor. Thr 41 was not identified in the spectrum, but Ser 40 and residues 42 and 43 show significant shift changes and decrease in intensity on addition of nicotinate. Residues 120–129 are in helix F, and the largest ^15^N shift changes in the protein are at residues 122, 124 and 125, at or near Phe 124, the residue directly contacting the nicotinate. Glu 165 and Gly 166 also form direct contacts with the ligand and show the largest weighted shift changes overall and large decreases in peak intensity. Leu 203 and Thr 202 show significant ^15^N shifts, but little shift in ^1^H and hence a small weighted overall shift, however Asn 200, which contacts Thr 202, among other residues, shows a larger ^1^H shift, giving a larger weighted overall shift on binding ligand, despite having a small change in ^15^N shift. The other significant shifts are of Trp 138, which is in the active site and contacts Glu 165 via the indole NH, and Val 188, which contacts Ile 164.

While the largest weighted overall shift changes in helix F are at residues 126 and 127, most of the residues of this helix, those from 116 to 129, show significant shifts in ^1^H or ^15^N. This suggests that this helix is affected by more than just direct interaction with the ligand, possibly a change in mobility as there is no change in the crystal structure of the protein with ligand. These residues are in the region with higher B factors in the crystal structure of the free protein.

Other residues that show a significant shift only in ^15^N on ligand binding include Tyr 68 and Asn 71 which are both close to Gly 166, while Ala 65 and Phe 70, which also shift, are close to Tyr 68. Tyr 68 is close to Phe 124 on the opposite subunit, the residue that contacts the ligand directly. The side chains of Phe 124 and Tyr 68 are likely to control the entry of the ligands into the active site of the protein (Fig. 5). A wide range of mutations at both of these amino acids, as well as at Phe 70, enhance the activity of the protein for the bulky prodrug CB1954 (Grove et al. 2003; Race et al. 2007). The residues Glu 165, Trp 138 and Ser 40 have recently been proposed to be important in catalysis in *Enterobacter cloacae* nitroreductase, in stabilising a bound water molecule in the correct orientation for donation of a proton in the reduction mechanism, while His 128 binds the nitro group to be reduced (Christofferson 2020). The shift changes observed further from the direct site of binding may therefore reflect motions affecting ligand binding and ultimately catalysis.

## Conclusion

We present nearly complete backbone assignments of *E. coli* NfsB, a 48 kDa homodimeric flavoprotein. The secondary structure from the chemical shifts are in good agreement with the crystal structure of the free protein, and the NH resonances most affected by nicotinate binding are at the binding site of the ligand. Additional chemical shift changes on ligand binding are seen further from the immediate binding site which may reflect other motions. These studies lay the foundations for studies of the dynamics of the protein or of other ligands binding to the protein in solution.

## Data Availability

The backbone assignments of the free protein have been deposited in the BioMagResBank, ID 50476. The assignments of the amide resonances of the protein bound to nicotinate have been deposited under ID 50576.
